# Mobile and Connected Health Technology Needs for Older Adults Aging in Place: Cross-Sectional Survey Study

**DOI:** 10.2196/13864

**Published:** 2019-05-15

**Authors:** Jing Wang, Yan Du, Deidra Coleman, Michelle Peck, Sahiti Myneni, Hong Kang, Yang Gong

**Affiliations:** 1 School of Nursing The University of Texas Health Science Center at San Antonio San Antonio, TX United States; 2 Cizik School of Nursing The University of Texas Health Science Center at Houston Houston, TX United States; 3 School of Biomedical Informatics The University of Texas Health Science Center at Houston Houston, TX United States

**Keywords:** mobile health, connected health, wearable technology, remote monitoring, independent living, aging in place

## Abstract

**Background:**

An increasing number of mobile and wearable devices are available in the market. However, the extent to which these devices can be used to assist older adults to age in place remains unclear.

**Objective:**

This study aimed to assess older adults’ perceptions of using mobile and connected health technologies.

**Methods:**

Using a cross-sectional design, a total of 51 participants were recruited from a senior community center. Demographics and usage of mobile or wearable devices and online health communities were collected using a survey questionnaire. Descriptive statistics assessed usage of devices and online health communities. The Fisher exact test was used to examine the relationship between technology usage and having access to a smartphone.

**Results:**

The sample was primarily comprised non-Hispanic white (35/51, 69%), educated (39/51, 76% any college), and female (36/51, 71%) participants, with an average age of 70 (SD 8) years. All participants were insured and nearly all lived at home (49/51, 94%). A total of 86% (44/51) of the participants had heard of wearable health devices, but only 18 out of 51 (35%) had ever used them. Over 80% (42/51) expressed interest in using such devices and were interested in tracking exercise and physical activity (46/51, 90%), sleep (38/51, 75%), blood pressure (34/51, 67%), diet (31/51, 61%), blood sugar (28/51, 55%), weight (26/51, 51%), and fall risk (23/51, 45%). The greatest concerns about using wearable devices were cost (31/51, 61%), safety (14/51, 28%), and privacy (13/51, 26%); one-fourth (12/51) reported having no concerns. They were mostly interested in sharing data from mobile and connected devices with their health care providers followed by family, online communities, friends, and no one. About 41% (21/51) of the older adults surveyed reported having ever heard of an online health community, and roughly 40% (20/51) of the participants reported being interested in joining such a community. Most participants reported having access to a smartphone (38/51, 74%), and those with such access were significantly more likely to show interest in using a wearable health device (*P*<.001) and joining an online health community (*P*=.05).

**Conclusions:**

Our findings suggest that, although few older adults are currently using mobile and wearable devices and connected health technologies for managing health, they are open to this idea and are mostly interested in sharing such data with their health care providers. Further studies are warranted to explore strategies to balance the data sharing preference of older adults and how to best integrate mobile and wearable device data with clinical workflow for health care providers to promote healthy aging in place.

## Introduction

### Background

Aging in place is referred as being able to continue living in one’s own residence as they age [1]. According to the American Association of Retired Persons [2], nearly 90% of older adults would like to remain in their home for as long as possible [2]. However, aging is accompanied with a variety of chronic conditions, and aging in place is challenged by physical and cognitive function decline and consequently the lack of independence [3]. An increasing number of mobile, wearable, and remote monitoring devices, as well as online health communities, have shed light on aging in place and are making it increasingly viable for older adults to age at home.

In today’s market, there are numerous available and emerging tech products which can help older adults age in place, including but not limited to smart-home sensors, smartphone applications, wearable trackers, remote monitoring devices, online health communities, and telehealth platforms [4]. Wearable trackers and telehealth platforms can help the elderly monitor and manage their chronic diseases and improve health outcomes. There is evidence in the literature that wearable trackers and telehealth platforms can help the elderly engage in a healthy lifestyle [5,6], adhere to medication regimens [5,7], and monitor biomarkers and health indicators from home [8,9]. All these data can be shared with their health providers electronically and help them to manage chronic diseases and improve health outcomes.

In addition, the tremendous function of these technologies provides opportunities for older adults to safely and effectively perform activities of daily living, as well as instrumental activities of daily living. For instance, smart-home sensors embedded in walls, ceilings, beds, and floors can detect motion, gait, and fall risk and can help prevent injures [10]; mobile apps can track an older adult’s location, sending a signal to their own or their children’s smartphone app [11,12]. Digital memory aids can help the elderly remember chores or errands and remind them to pay bills [13].

Furthermore, smartphone and online health communities enable older adults get connected and interact with others [14,15]. Social interaction and social engagement are important factors for aging in place [16,17] and have been widely reported to be positively associated with healthy behaviors, self-reported health, physical function, cognitive function, psychological well-being, and longevity, even for the oldest old [14,18-21]. There are several possible underlying mechanisms for the positive effects of smartphones and online health communities on older adults’ overall well-being. For instance, online health communities provide older adults a platform to seek health information [22]. Also, the leisure activity and expanded social network available through online communities may provide social support, contribute to self-preservation, and serve as an opportunity for self-discovery and growth [14].

Mobile and connected health technologies can help older adults improve health conditions, slow down functional decline, ensure safety, stay connected, and maintain the ability and capacity to live independently. However, studies have documented that the usage of technology is low in older adults [23], especially compared with younger age groups [24], and the major reasons for using technology in the elderly are email communication, search engines, text processing, and Web-based shopping [25]. The acceptance and usage of mobile and connected technology in the elderly are determined by a variety of factors, including but not limited to person-related, technology-related, and contextual barriers [26,27].

### Objectives

However, the extent to which mobile and connected health technologies can be used to assist older adults to age in place has not been well defined. Therefore, we aimed to study the older adults’ perspectives of using mobile, wearable, and remote monitoring devices, as well as online health communities for aging in place.

## Methods

### Study Design and Study Population

Using a cross-sectional design, a total of 51 participants were recruited from a senior community center in Harris County, Texas, in 2016 to assess older adults’ perceptions of using mobile, wearable, or remote monitoring technology, as well as online health communities. After completing a community assessment which included conducting a windshield survey, interviewing stakeholders within the senior community center, and interviewing older adult residents, along with a systematic review of currently available mobile apps, wearable trackers, and personal health devices, we developed a survey to examine if older adults would be interested in using mobile and wearable health devices as a means of tracking their health, as well as using online health communities.

The eligibility criteria for enrolling in the senior center included (1) residing in Harris County, Texas, (2) being aged 55 years or older, and (3) signing a participant agreement that includes a release of liability and acceptance of precinct and county rules concerning the operation of the center [28]. All seniors who registered in the senior center were invited to participate in the study. Those who agreed to participate in the study were recruited. Consent forms were obtained from each participant before conducting any study activities. The Institutional Review Board of the University of Texas Health Science Center at Houston approved this study.

### Data Collection

All data were collected using survey questionnaires. Demographic characteristics included age in years, gender, race/ethnicity, education (college and higher vs other), marital status, insurance status, and living arrangement. A brief introduction and description of wearable devices were included in the questionnaire. Pictures demonstrating a set of wearable devices were also included in the questionnaire to help older adults recognize different types of health technology devices. Similarly, a statement describing online health communities was provided in the questionnaire. Questions were asked to assess older adults’ usage of home health monitoring devices, wearable health devices, and online health communities. For example, “Have you ever used a wearable health device?” was asked to assess the usage of wearable health devices. Available options for this question were “Yes” and “No.” We further explored their access to technological devices, their concerns of using health technologies, future interest in using wearable health devices and online health communities, the preference of health information they would like to track and share, and with whom they would prefer to share health information. For instance, “Who would you like to connect with in an online community? (Select ALL that apply)” was asked to assess their using of online health community. Available answers for the question included *family*, *friends*, *caregiver*, *health care provider*, *people in similar situations as me whom I don’t know*, *other (please specify)*, and *none of the above*.

### Data Analysis

Descriptive analysis was used to examine the demographics of the sample. The same analytic approach was conducted to assess the accessibility, usage, data sharing preference, and concerns when using wearable devices or online health communities. Mean (SD) and number (percentage), wherever appropriate, were used to describe the results. Given the small sample size, the Fisher exact test [29] was adopted to assess the relationship between accessibility and the usage of wearable devices/online health communities. We examined this relationship as smartphones can be used as both a mobile device and as a means to access online health communities [30], which can better help us understand the feasibility of promoting health technologies in older adults.

## Results

### Sample Characteristics

The sample was primarily comprised non-Hispanic white (35/51, 69%), educated (39/51, 76% of any college), and female (36/51, 71%) participants, with an average age of 70 (SD 8) years (Table 1). All participants were insured, and 47% (24/51) reported having private insurance. Nearly all participants lived at home (41/51, 94%) and none lived in an assisted living facility or nursing home. About 27% (11/51) lived alone, and the rest were living with a family or another person.

### Mobile, Wearable, and Remote Technology Use in This Sample

About 43 out of 51 (84%) of the study participants used a health monitoring device at home (Table 2) and 47% (24/51) of them used paper to write down the results, 29% (15/51) did not keep a record, only 10% (5/51) used a computer or phone to type the results, 6% (3/51) downloaded results, 6% (3/51) had results automatically transferred to a smartphone, and 6% (3/51) let health care providers download results when visiting doctors’ offices.

A total of 86% (44/51) of the participants had heard of wearable health devices but only 35% (18/51) had never used them. Over 80% (42/51) expressed interest in using such devices (Table 2) and were interested in tracking exercise/physical activity (46/51, 90%), sleep (38/51, 75%), blood pressure (34/51, 67%), diet (31/51, 61%), blood sugar (28/51, 55%), weight (26/51, 51%), and fall risk (23/51, 45%). The greatest concern about using wearable devices was cost (31/51, 61%), followed by safety (14/51, 28%) and privacy (13/51, 26%); approximately one-fourth of the sample (12/51, 24%) reported having no concerns.

Older adults reported feeling comfortable sharing exercise/physical activity, diet, sleep, heart rate, breathing, body posture, blood pressure, blood sugar, weight, mood, and fall risk data captured by mobile and wearable devices. They are mostly interested in sharing these data with their health care provider (30/51, 59% heart rate; 29/51, 57% blood pressure; 29/51, 57% exercise/physical activity; 28/51, 55% blood sugar; 27/51, 53% sleep; 27/51, 53% weight; 25/51, 49% diet) followed by family, online communities, friends, and no one (Table 3).

**Table 1:** Demographic characteristics of the sample (N=51).

Variables		Statistics
Age (years), mean (SD)		70 (8)
Gender (female), n (%)		36 (71)
**Race/ethnicity, n (%)**		
	Hispanics	4 (8)
	Non-Hispanic white	35 (69)
	Non-Hispanic black	7 (14)
	Non-Hispanic Asian	4 (8)
Education (college and higher), n (%)		39 (76)
Married (yes)^a^, n (%)		31 (61)
Private insurance (yes), n (%)		24 (47)
Live at home/retired home (yes), n (%)		49 (94)
Live alone (yes), n (%)		14 (28)

^a^Never married, widowed, and divorced were considered as not married.

**Table 2:** Summary for mobile, wearable, remote technology use in this sample (N=51).

Health technology usage	Statistics, n (%)
Ever used an HMD^a^ at home (yes)	43 (84)
Ever heard of WHD^b^ (yes)	44 (86)
Ever used a WHD^b^ (yes)	18 (35)
Currently using a WHD^b^ (yes)	13 (26)
Be interested in using a WHD^b^ (yes)	42 (82)
Ever heard of OHC^c^ (yes)	21 (41)
Be interested in joining an OHC^c^ (yes)	20 (39)

^a^HMD: health-monitoring device.

^b^WHD: wearable health device.

^c^OHC: online health community.

**Table 3:** Preference of sharing data captured by mobile and wearable devices in this sample (N=51).

Health data	Health care providers, n (%)	Family, n (%)	Friends, n (%)	Online health communities, n (%)	No one, n (%)	Other groups, n (%)
Exercise/physical activity	29 (57)	23 (45)	14 (28)	14 (28)	4 (8)	1 (2)
Diet	25 (49)	17 (33)	9 (18)	11 (22)	7 (14)	0 (0)
Sleep	27 (53)	19 (37)	9 (18)	10 (20)	6 (12)	0 (0)
Heart rate	30 (59)	18 (35)	6 (12)	8 (16)	3 (6)	0 (0)
Breathing	21 (41)	14 (28)	5 (10)	5 (10)	5 (10)	0 (0)
Body posture	18 (35)	12 (24)	3 (6)	5 (10)	7 (14)	0 (0)
Blood pressure	29 (57)	18 (35)	6 (12)	9 (18)	3 (6)	0 (0)
Blood sugar	28 (55)	15 (29)	5 (10)	8 (16)	3 (6)	0 (0)
Weight	27 (53)	14 (28)	6 (12)	8 (16)	5 (10)	0 (0)
Mood	18 (35)	12 (24)	5 (10)	6 (12)	11 (22)	0 (0)
Fall risk	20 (39)	11 (22)	3 (6)	6 (12)	8 (16)	0 (0)
Other	3 (6)	1 (2)	1 (2)	1 (2)	0 (0)	0 (0)

### Online Health Communities

About 41% (21/51) of the older adults surveyed reported having ever heard of an online health community (Table 2). In addition, roughly 40% (20/51) of the participants reported that they would be interested in joining such a community, and the same percentage reported not being sure if they would join; 47% (24/51) of the participants surveyed reported that they would like to use the online community to connect to other people in similar situations who they do not know or to their health care provider (21/51, 41%). The data that older adults were mostly interested in sharing with other people in the online health communities were exercise/physical activity data (14/51, 28%), diet (11/51, 22%), and sleep (10/51, 20%). Finally, the most common concerns about joining an online health community was privacy (28/51, 55%) and fraud (20/51, 39%). However, about 20% (10/51) of all the participants surveyed reported having no concerns about joining online health communities.

### Smartphone Access and Usage of Mobile/Connected Health Technology

Most participants reported having access to a smartphone (38/51, 74%; Table 4) and those with such access were significantly more likely to show interest in using a wearable health device (*P*<.001), despite the fact that they were not more likely to be currently using a wearable health device (*P*=.14) or to have previously used a wearable health device (*P*=.33) or a remote monitoring device at home (*P*=.18). In addition, those having access to a smartphone were more likely to report interest in joining an online health community (*P*=.05).

**Table 4:** The Relationship between health technology usage and having access to a smartphone in this sample (N=51).

Health technology usage	Having access to a smartphone		*P* value
	Yes (n=38)	No (n=13)	
Ever used an HMD^a^ at home (yes)	34 (90)	9 (69)	.18
Ever heard of WHD^b^ (yes)	32 (87)	12 (92)	>.99
Ever used a WHD (yes)	15 (41)	3 (23)	.33
Currently using a WHD (yes)	12 (32)	1 (8)	.14
Be interested in using a WHD (yes)	36 (97)	6 (50)	<.001
Ever heard of OHC^c^ (yes)	18 (47)	3 (27)	.31
Be interested in joining an OHC (yes)	18 (48)	2 (15)	.05

^a^HMD: health-monitoring device.

^b^WHD: wearable health device.

^c^OHC: online health community.

## Discussion

### Principal Findings

This study explored to what extent mobile and connected health technologies can be utilized by older adults for aging in place. Overall, our findings revealed that there is a potential to promote mobile and connected health technologies in the elderly to improve health, extend independent life span, and age at home. However, some top concerns of using health technologies need to be addressed, such as the high cost for wearable health devices and privacy and fraud issues for using online health communities. One of our key findings was that a majority of older adults were interested in tracking health information and most were interested in sharing tracked health information with their health care providers, which imply the potential for connecting data from mobile and wearable devices to clinicians to promote aging in place.

Our study found that health technology use in older adults was low, and the top concerns for using wearable health devices and online health communities were different. Previous studies have reported that general technology use in older adults is relatively low (eg, 40.0% for email and texting and 42.7% for internet use), especially compared with younger groups [31]. We found that usage of wearable health devices was even lower in older adults (18/51, 35% for ever used; 13/51, 26% for currently using). The study shows that the top concern of using wearable health devices among older adults was the cost. This is partially consistent with Peek et al’s study, who reviewed 16 articles and reported that one of the major concerns of using technology for aging in place in community-dwelling older adults is the high cost [32]. However, a majority of the studies included in the review were qualitative studies, and they only examined the overall technology usage or focused solely on one specific health technology tool (eg, memory aids). Our study has quantified the concerns and provided a more practical implication. For example, for wearable health devices, the top concern was cost, whereas for online health communities, the top concerns were privacy and fraud. Therefore, we suggest that researchers, manufacturers/marketers, and policy makers work together and address one of the top concerns of using wearable health devices in the elderly. For instance, free wearable devices might promote wearable health device usage in the older population, and a free wearable health device intervention might affect health outcomes and promote healthier aging in place. Furthermore, studies are needed to assess whether this line of interventions would be cost-effective and reduce medical costs in the long term. Meanwhile, the usage of online health communities in older adults is increasing, but older adults are less knowledgeable about internet security than younger adults, and they are more susceptible to internet fraud [33]. However, effective and feasible strategies to increase safe internet usage in older adults, considering their knowledge and cognitive function, are still limited [34]. We call for the development of technologies and training materials to educate older adults in Web-based safety.

Importantly, this study found that a majority of older adults were interested in using wearable health devices, over one-third were interested in joining an online health community, and those having access to a smartphone showed higher interest in using wearable health devices and joining in an online health community. The findings reveal that there is a potential to promote use of health technologies in older adults to help them age in place. First, our findings are in line with the technology acceptance model (TAM). The major components of TAM consist of external variables, perceived usefulness, perceived ease of use, attitude, behavioral intention, and actual use [35,36]. External variables would refer to factors such as cost and fraud [37], which have been discussed above. Those who have access to a smartphone might perceive greater ease of use and usefulness of health technologies [38]; and consequently, as being found in our study, having access to a smartphone was positively related to the interest of using wearable health devices and joining in an online health community. Our findings provide critical implications for promoting health technology use in older adults. Specifically, the intervention strategies for those who have access to a smartphone might be different than for those who do not have such access. Further studies might be indicated to explore the specific needs of older adults using health technologies based on their previous experience of using a smartphone. However, a previous study has reported that those who have access to a smartphone tend to be younger, with higher education and income [24], all of which are highly related to the usage of technology [31]. Given the small sample size of our study, we were unable to determine whether the positive associations between having access to a smartphone and interest in using wearable devices or online health communities were confounded by demographic factors. Future studies with a larger sample size are needed to clarify the real underlying reasons of this positive association and provide further implications for intervention.

Notably, our findings contribute to understanding preferences for health information sharing in older adults. We found that if wearable health devices were available for older adults, a majority of them would be interested in tracking physical activity, sleep, diet, blood pressure, blood sugar, and weight, and they felt more comfortable sharing this information with their health providers than with their families, friends, or others. Even for online health communities, they would want to connect with their providers. This has significant implications for our future intervention strategies. Given the advantages of using mobile and connected health technologies for tracking health information and facilitating chronic condition monitoring and management [8], older adults’ interest in tracking health information and sharing it with health providers shows promise for the promotion of aging in place. To our knowledge, this is the first study to explore preferences for sharing health information in older adults comprehensively. Overall, studies examining the sharing preference of tracked health information are scarce. Available literature has documented older adults’ preference of sharing health information with researchers and families [39,40]. For data sharing preference with health providers in older adults, previous studies have not achieved a conclusion. One study found that a majority of older adults were willing to share monitored health information with the family or one’s doctor, but it did not differentiate sharing preference between family and health providers and only focused on those aged 80 and over [41]. Another study reported that compared with those aged 18 to 24 years, older adults aged 65 and over were less comfortable sharing mobile health information with health care providers; however, that study did not examine older adults’ willingness to share mobile tracked health information with providers [42]. Our findings indicate that connecting older adults with health care providers to help them age in place is possible. However, it should be noted that previous study shows that formal caregivers and health care providers would like their older clients to track health information for self-care and they would also be interested in providing feedback and individualized care to older adults based on data recorded by wearable health devices, while they were actually less interested in reviewing such massive data [43]. Therefore, studies are warranted to explore strategies to balance the data sharing preference of older adults and the availability of health care providers and effectively use monitored data to improve health and promote aging in place. For example, it would be beneficial to explore how to use informatics technologies to generate health implications from tracked data, so that health providers can use tracked data and provide health care recommendations, without the need to manually review massive amounts of data.

This study may be limited by the small sample size. Even though we did cover a variety of racial/ethnic populations, most of the study participants were non-Hispanic white. Notably, this study was conducted in Harris County, Texas [44], with approximately 43% Hispanic population, whereas in our study, only 8% were Hispanic. Therefore, the findings of the study may not be generalizable to the general population in Harris County. Future studies with a larger sample size and a focus on greater recruitment of minority groups are warranted. The literature has extensively documented the facilitators and challenges of using technology in the elderly; future translational research studies should consider a comprehensive approach to promote mobile and connected health technology in older adults, combining technical, informational, and social support [45].

### Conclusions

The study results show that while many older adults were not currently using mobile and wearable devices, or online health communities, they were open to this idea, especially among those who had access to a smartphone. Older adults were most interested in sharing data captured by mobile and wearable devices with their health care providers. Similarly, health care providers were the preferred recipients of health care information sharing in online health communities. These findings confirm previous studies on technology acceptance and adoption by older adults. Furthermore, this study reveals the possibility of promoting mobile and connected health engagement in older adults. Researchers, technology manufacturers/marketers, policymakers, and health providers need to make efforts to increase accessibility and safety of using mobile and connected health technology. Further studies are warranted to explore strategies to balance the data sharing preference of older adults and how to best integrate mobile and wearable device data within the clinical workflow for health care providers to promote healthy aging in place.

## Abbreviations

TAM: technology acceptance model
